# NLGP counterbalances the immunosuppressive effect of tumor-associated mesenchymal stem cells to restore effector T cell functions

**DOI:** 10.1186/s13287-019-1349-z

**Published:** 2019-09-23

**Authors:** Tithi Ghosh, Partha Nandi, Nilanjan Ganguly, Ipsita Guha, Avishek Bhuniya, Sarbari Ghosh, Anirban Sarkar, Akata Saha, Shayani Dasgupta, Rathindranath Baral, Anamika Bose

**Affiliations:** Department of Immunoregulation and Immunodiagnostics, Chittaranjan National Cancer Institute (CNCI), 37, S. P. Mukherjee Road, Kolkata, 700026 India

**Keywords:** Cystathionase, Dendritic cells, IL-10, Mesenchymal stem/stromal cells, Neem leaf glycoprotein, Tumor microenvironment, T cells

## Abstract

**Background:**

A dynamic interaction between tumor cells and its surrounding stroma promotes the initiation, progression, metastasis, and chemoresistance of solid tumors. Emerging evidences suggest that targeting the stromal events could improve the efficacies of current therapeutics. Within tumor microenvironment (TME), stromal progenitor cells, i.e., MSCs, interact and eventually modulate the biology and functions of cancer and immune cells. Our recent finding disclosed a novel mechanism stating that tumor-associated MSCs inhibit the T cell proliferation and effector functions by blocking cysteine transport to T cells by dendritic cells (DCs), which makes MSCs as a compelling candidate as a therapeutic target. Immunomodulation by nontoxic neem leaf glycoprotein (NLGP) on dysfunctional cancer immunity offers significant therapeutic benefits to murine tumor host; however, its modulation on MSCs and its impact on T cell functions need to be elucidated.

**Methods:**

Bone marrow-derived primary MSCs or murine 10 T1/2 MSCs were tumor-conditioned (TC-MSCs) and co-cultured with B16 melanoma antigen-specific DCs and MACS purified CD4^+^ and CD8^+^ T cells. T cell proliferation of T cells was checked by Ki67-based flow-cytometric and thymidine-incorporation assays. Cytokine secretion was measured by ELISA. The expression of cystathionase in DCs was assessed by RT-PCR. The STAT3/pSTAT3 levels in DCs were assessed by western blot, and STAT3 function was confirmed using specific SiRNA. Solid B16 melanoma tumor growth was monitored following adoptive transfer of conditioned CD8^+^ T cells.

**Results:**

NLGP possesses an ability to restore anti-tumor T cell functions by modulating TC-MSCs. Supplementation of NLGP in DC-T cell co-culture significantly restored the inhibition in T cell proliferation and IFNγ secretion almost towards normal in the presence of TC-MSCs. Adoptive transfer of NLGP-treated TC-MSC supernatant educated CD8^+^ T cells in solid B16 melanoma bearing mice resulted in better tumor growth restriction than TC-MSC conditioned CD8^+^ T cells. NLGP downregulates IL-10 secretion by TC-MSCs, and concomitantly, pSTAT3 expression was downregulated in DCs in the presence of NLGP-treated TC-MSC supernatant. As pSTAT3 negatively regulates cystathionase expression in DCs, NLGP indirectly helps to maintain an almost normal level of cystathionase gene expression in DCs making them able to export sufficient amount of cysteine required for optimum T cell proliferation and effector functions within TME.

**Conclusions:**

NLGP could be a prospective immunotherapeutic agent to control the functions and behavior of highly immunosuppressive TC-MSCs providing optimum CD8^+^ T cell functions to showcase an important new approach that might be effective in overall cancer treatment.

**Electronic supplementary material:**

The online version of this article (10.1186/s13287-019-1349-z) contains supplementary material, which is available to authorized users.

## Background

Early molecular genetic studies delineated tumors arising from normal cells through genetic alterations hampering the tightly regulated systems for growth control while almost entirely overlooking the vital contribution of tumor stroma. However, it is being increasingly reported that tumor cells interact with their microenvironment or the stroma ensuing tumor growth and progression [1]. Basically, stroma is composed of tumor-promoting and tumor-opposing cells including fibroblasts/myofibroblasts, glial cells, epithelial cells, adipocytes, immune cells, vascular cells, smooth muscle cells along with soluble molecules, and extracellular matrix components. While none of these cells are themselves malignant, due to their interactions with each other, and with cancer cells directly or indirectly, they acquire an abnormal phenotype and altered functions [2].

In addition to mature stromal elements affecting carcinogenesis, attention has now shifted to the progenitor cells of the stroma, the mesenchymal stem cells (MSCs). MSCs are recruited to the site of tissue injury for repairing, maintaining tissue homeostasis, and imparting immunomodulation. Similar functions are displayed by MSCs during tumor development. The interaction between MSCs and cancer cells has been found to involve two unique properties: their immunosuppressive effect and their ability to migrate. Their immunomodulatory effects are a key factor that influences tumor development [3]. MSCs display suppressive effects on both innate and humoral immunity by inhibiting T cell proliferation [4], dendritic cell (DC) maturation [5], and natural killer (NK) and B cell activation [6, 7], while simultaneously increasing the production of regulatory T cells (Tregs) [8].

In this context, we have recently reported a novel mechanism that tumor-associated MSCs promote immune-evasion, targeting the priming and expansion of naïve T cells by blocking cysteine export from DCs. In particular, tumor-associated MSCs repress cystathionase gene expression on DCs and thereby inhibit cysteine production to export it out to neighboring T cells required for their optimum proliferation [9]. Therefore, modulating the immunosuppressive nature of MSCs in progressive tumor has potential clinical applications in terms of cancer management. We have consistently reported murine tumor growth restriction by a natural, non-toxic immunomodulator, neem leaf glycoprotein or NLGP [10], by robust immunomodulation [11, 12]. Moreover, NLGP was reported to have therapeutic efficacy to restrict the growth of established murine sarcoma and melanoma by activating CD8^+^ T cell responses [13–15] within NLGP normalized TME [14].

In the present study, we showed how NLGP modulates tumor-associated MSCs by reducing their IL-10 secretion that ultimately results in reduced phosphorylation of STAT3 leading to enhanced transcription of cystathionase in DCs. Consequently, DCs are able to export out sufficient amount of cysteine to the cognate T cells required for their optimum proliferation and late-phase effector function. Accordingly, restricted tumor growth was observed following adoptive transfer of T cells rescued from the negative influence of TC-MSCs by NLGP.

## Materials and methods

### Neem leaf glycoprotein (NLGP)

Extract from neem (*Azadirachta indica*) leaves was prepared by the method as described previously [16]. Mature leaves of the same size and color (indicative of same age), taken from a standard source, were shed-dried and pulverized. Leaf powder was soaked overnight in phosphate-buffered saline (PBS), pH 7.4. Supernatant was collected by centrifugation at 1500 rpm, extensively dialyzed against PBS, pH 7.4, and concentrated by Centricon membrane filter (Millipore Corporation, MA, USA) with 10 kDa molecular weight cut-off. Purified NLGP was checked for its quality by electrophoresis and HPLC using routine laboratory methods. Biological activity of purified NLGP was checked by tumor growth restriction assay before use. The protein concentration was measured using Folin-phenol reagent as described [17].

### Cells and cell line culture

Primary MSCs were isolated from murine bone marrow and culture-expanded as described earlier [9]. The murine MSC line C3H10T1/2 (CCL-226) cells were obtained from ATCC (Manassas, VA) and grown in DMEM (Gibco, Grand Island, NY) supplemented with 10% FBS, 233.6 mg/ml glutamine, 25 mM glucose, and 80 U/ml penicillin/streptomycin, according to the provider’s recommendation in a humidified atmosphere of 5% CO_2_ at 37 °C.

Tumor conditioning of C3H/10 T1/2 cells (TC-MSC) was accomplished by exposing MSC cells to B16 melanoma confluent culture supernatants in hypoxia (37 °C at 1–2% O_2_ overnight in Hypoxia Chamber- Stem Cell Technologies, Vancouver, BC, Canada).

B16F10 murine melanoma cells (B16 melanoma) were obtained from the National Center for Cell Sciences, Pune, India. They were maintained in complete DMEM high glucose media supplemented with 10% (*v*/*v*) heat inactivated FBS, 2 mM l-glutamine, 100 U/ml penicillin, and 100 μg/ml streptomycin at 37 °C with the supply of 5% CO_2_.

### Mice and tumor

Inbred C57BL/6 (H-2b) female mice (age, 4–6 weeks, body weight, 21 g on average) were procured from Animal Facilities of the National Institute of Nutrition (Hyderabad, India). An autoclaved dry pellet diet (Epic Laboratory Animal Feed, Kalyani, India) and water were provided ad libitum. All experiments in this study were performed in accordance with the guidelines established by the Institutional Animal Care and Ethics Committee (Approval No. IAEC-1774/RB-2(Extn)/2016/12).

Solid melanoma tumors were developed in mice by inoculation B16 melanoma F10 cells (2 × 10^5^) subcutaneously into syngenic mice and allowed to grow as solid tumor. Area of solid tumor (in mm^2^) was monitored weekly by caliper measurement using the formula (width × length).

### Generation of bone marrow-derived DCs

DCs were generated from bone marrow (BM) precursors of C57BL/6 mice, as described [18]. In brief, single-cell suspension was obtained after flushing bone marrow from the tibia and femurs. Erythrocytes were lysed by re-suspending the cell pellet in a hypotonic buffer. The cells were cultured (2 × 10^6^ cells/well) with complete RPMI 1640 medium containing 10% FBS, 10 ng/ml rm-GMCSF, and 5 ng/ml rm-IL-4. On day 6 of culture, nonadherent cells obtained from these cultures were considered to be immature BMDC.

On day 8, cells were harvested and positively selected with CD11c magnetic beads (Miltenyi Biotech, BergischGladbach, Germany) and matured by addition of LPS (1 μg/ml; Sigma-Aldrich, USA) to cultures for 48 h at 37 °C. Immature and LPS-matured DCs were 85 to 90% CD11c^+^ and expressed MHC II and CD86 as analyzed by flow cytometry (data not shown). Antigen-loading of day 8 BMDCs (1 × 10^6^ cells/ml) was accomplished by incubation with B16 melanoma antigen (5 μg/ml of culture media) overnight at 37 °C. Semi-adherent cells were then collected and considered as melanoma antigen-pulsed BMDCs.

### Antibodies

Anti-CD4/CD8 magnetic particles, anti-mKi-67, anti-mTGFβ antibodies, anti-m vimentin, pSTAT3, and all recombinant antibodies were purchased from BD Biosciences (San Jose, CA). Antibodies against mIL-6 and mIL-10 were purchased from Biolegend (San Diego, CA). Antibodies against mIFNγ and mCD105 were obtained from eBiosciences (San Diego, CA), while anti-mVEGF was purchased from Santa Cruz Biotechnology (Dallas, TX). Used antibodies in detail with catalog number, clones, and isotypes are presented in Additional file 2: Table S1.

### Isolation of T lymphocytes

After harvesting spleens and lymph nodes from mice, tissues were mechanically disrupted into single-cell suspensions and filtered through sterile 75-mm nylon mesh. Filtered cells were collected, then overlaid on lymphocyte separation medium (MP Biomedicals, Solon, OH) and centrifuged for 30 min at 800×*g*. The mononuclear layer was collected from the medium interface and resuspended in complete RPMI 1640 media (Invitrogen, Camarillo, CA). Splenic CD4^+^ and CD8^+^ T cells were then positively selected using BD iMag anti-m CD4 and CD8 Particles – DM (BD Biosciences, San Diego, CA). Flow-cytometric analyses confirmed the purity of cells to be > 95%. T cells were cultured either in complete RPMI 1640 medium (Invitrogen, Camarillo, CA) or in cystine/methionine free medium (CELLClone, Genetix, New Delhi, India) as indicated in text.

### Cytotoxicity assay

Cytotoxicity of CD8^+^ T cells against mouse melanoma cells was determined by measuring lactate dehydrogenase (LDH) released by target cells using a commercially available kit (Roche Diagnostics, Mannheim, Germany).

### RT-PCR

Cellular RNA was isolated using TRIzol (Invitrogen, Camarillo, CA) and random hexamers used to generate corresponding cDNA (First Strand cDNA Synthesis Kit; Fermentas, Hanover, MD). Amplification was performed using 23 Bio Mix Red (Bioline, Tauntan, MA) with the following program: 94 °C for 5 min; 35 cycles of 94 °C for 30 s, 54–57 °C for 30 s, and 72 °C for 1 min; and 72 °C for 5 min. PCR products were identified by image analysis software for gel documentation (Versadoc; BioRad Laboratories, Hercules, CA) after electrophoresis on 1.5% agarose gels and staining with ethidium bromide (Sigma-Aldrich, USA). RT-PCR primers were designed and purchased from MWG-Biotech (Bangalore, India).

### Flow-cytometric staining

Flow cytometric analysis for cell-surface phenotype was performed after the staining of cells (1 × 10^6^) with fluorescently labeled antibodies (specific and isotype-matched controls) as per the manufacturer’s recommendations. After incubation for 30 min at 4 °C in the dark, labeled cells were washed twice with FACS buffer (0.1% BSA and 0.05% sodium azide in PBS) before being analyzed by flow cytometry. Similarly, intracellular molecules (i.e., IFNγ) were stained with anti-mouse fluorescence-labeled antibodies using Cytofix/Cytoperm reagents per the manufacturer’s instructions (BD Biosciences, San Diego, CA). For Ki67 staining, cells were pelleted by centrifugation, and 70–80% chilled ethanol was added to fix the pellet (1–5 × 10^7^ cells) with vortexing, before a subsequent incubation at − 20 °C for 2 h. Fixed cells were then washed twice with staining buffer and centrifuged for 10 min at 200×*g*, before being diluted to a concentration of 1 × 10^7^ cells/ml for staining and corollary flow cytometry analyses.

For all immunofluorescence analyses, cells were fixed with 1% paraformaldehyde in PBS, and acquisition was performed using a FACSCalibur (Becton Dickinson, NJ, USA). Data was analyzed with Cell Quest software. Suitable negative isotype controls were used to establish background staining profiles. The percentage of positively stained populations was determined using quadrant statistics established using FlowJo software (Tree Star, Ashland, OR).

### Cytokine detection assay

Secreted cytokines like IL-6, IL-10, VEGF, and TGFβ in MSC/TC-MSC culture supernatants and IFNγ and IL-2 in the supernatants of DC-T cell-MSC co-cultures were measured by ELISA using commercial kits (OptEIA, BD Pharmingen, San Diego, CA), and optical density was measured at 450 nm using microplate reader (BioTek Instruments Inc., Vermont, USA).

### Treatment with recombinant cytokines

To study the effect of cytokines, cells were treated with recombinant murine cytokines (250–1000 ng/ml/10^6^ cells) for 24–48 h at 37 °C in 5% CO_2_. After incubation, the cells were washed three times with FBS-free DMEM prior to experimental use.

### Lymphocyte proliferation

Isolated CD4^+^ and CD8^+^ T cells (5 × 10^5^/well) were co-cultured with mitomycin C (80 mg/ml for 1 h)-treated DCs (2 × 10^5^/well) in the presence or absence of MSC or TC-MSC-derived culture supernatants with or without NLGP for 3 days in 96-well plates, with each test performed in triplicate. Assay wells containing no DCs and T cells treated with ConA were used as negative and positive proliferation controls, respectively. After 72-h incubation at 37 °C, 20 μl (0.5 mCi) [^3^H] thymidine was added to each well and plates were incubated for an additional 24 h at 37 °C in 5% CO_2_. Cells were harvested with a cell harvester (PerkinElmer, Waltham, MA) and analyzed for uptake of radioactivity on a b-scintillation counter (PerkinElmer, Waltham, MA).

### Si-RNA-mediated STAT3 silencing

BMDCs were cultured up to 70% confluency. STAT3-specific Si-RNA (Santacruz Biotechnology, Dallas, TX) was then added in DC culture to a final concentration of 100 nM. Fifty micromolar Si-RNA (25 μl) and 6 μl of Lipofectamine were added to two Opti-MEM aliquots of 250 μl each and incubated for 5 min at RT. Then, the Si-RNA/Opti-MEM and the Lipofectamine/Opti-MEM (500 μl total volume) were mixed and allowed to incubate for 20 min at RT. Si-RNA-containing medium was then added to the DC culture. Finally, expression of stat3 was checked both in untreated and siRNA-transfected DCs by PCR analysis.

### Cell lysis and Western blot

Cell lysates were prepared by incubating cells from different treatment groups with RIPA buffer for 30 min and clarified by centrifugation. Protein concentrations of cell-lysate were determined [17]. The cellular lysate (protein concentration, 30–50 μg) was separated on 12% SDS-PAGE and transferred onto a PVDF membrane (Millipore, USA) using the BioRad Gel Transfer system. The membrane was first blocked with the 5% BSA for 2 h at room temperature. This was followed by incubation overnight at 4 °C with the primary antibody, then, with peroxidase-conjugated secondary antibody for 2 h at room temperature. Immunoreactive proteins were detected by addition of the HRP color development reagent according to the manufacturer’s protocol. The membrane was immersed into the solution for 1 min, wrapped with a Saran wrap, exposed to X-ray film, and developed, as described [19].

### Therapy with TC-MSC-educated T cells

TC-MSC or NLGP-modulated TC-MSC-educated CD8^+^ T cells (1 × 10^6^ cells/mice) were adoptively transferred to two groups of melanoma bearing mice respectively (tumor volume approximately 18 mm^2^) through tail vein, once in a week for 4 weeks in total. The third group of melanoma-bearing mice kept untreated as control. Tumor volume was measured by the formula mentioned above, and mouse survivability was checked by close monitoring.

### Statistical analysis

All reported results represent the mean ± SD of data obtained in six (as indicated for in vitro assays) independent experiments. Statistical significance was established by unpaired *t* test using INSTAT 3 Software (GraphPad Software), with differences between groups attaining a *p* value < 0.05 considered as significant.

## Results

### NLGP restores tumor-MSC-mediated inhibition of T cell functions

**Fig. 1:**
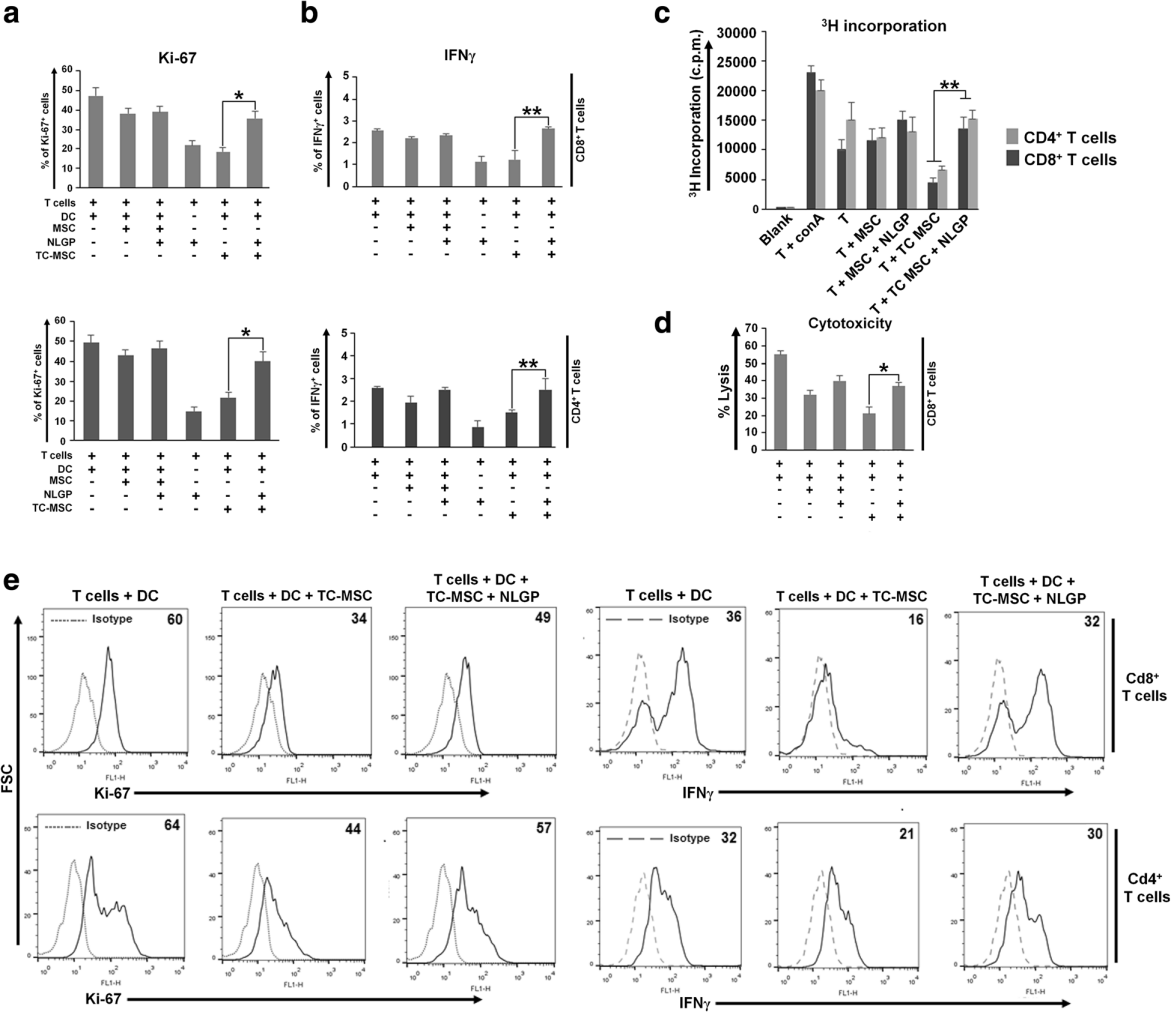
NLGP restores the TC-MSC-mediated inhibition of T cell proliferation and effector functions. T cells (CD8^+^ and CD4^+^) and DCs were isolated from naïve C57BL/6 mouse spleen and bone marrow, respectively, as described in the “Materials and methods” section. DCs and T cells (DC:T = 1:10) were cultured in the presence of either normal or TC-MSCs (MSC:T = 1:5) for 72 h, with or without NLGP or NLGP alone as indicated. **a** Expression of Ki-67 was analyzed on CD8^+^ and CD4^+^ T cells after 72 h by flow cytometry. Bar diagrams depict the mean ± SD of aggregate data obtained in six independent experiments performed. *p < 0.05; **b** IFNγ expression on T cells was analyzed by flow cytometry. Bar diagrams depict the mean ± SD of aggregate data obtained in six independent experiments performed. **p < 0.001; **c** Measurement of T cell proliferation after 72 h by [^3^H] thymidine incorporation assay. Bar diagrams show the mean ± SD of aggregate data obtained in six independent replicative experiments. *p < 0.05versus DC-induced T cells in the presence of TC-MSCs. **d** Purified CD8^+^ cells were cultured in the presence of either normal or TC-MSC for 72 h with or without NLGP. Cytotoxicity of these differentially exposed CD8^+^ T cells towards B16 melanoma cells was measured by LDH release assay. Aggregate data obtained from six independent experiments performed.*p < 0.05 versus DC-induced CD8^+^T cells in the presence of TC-MSC. **e** The expression of Ki67 and intracellular IFNγ on CD8^+^ and CD4^+^ T cells is depicted in representative histogram respectively

MSCs have the intrinsic property to home to the site of tumor development where T cells are present as a major executor of anti-tumor immunity. In this context, recently, we reported that within TME, tumor-conditioned MSCs (TC-MSCs) selectively suppress DC-induced T cell proliferation and late-phase effector functions [9]. Now, we are interested to see whether NLGP, a nontoxic natural immunomodulator, possess any ability to restore anti-tumor T cell functions by modulating TC-MSCs. In order to mimic the TME as much as possible in in vitro setting, we exposed MSCs to B16 melanoma tumor supernatant in a hypoxic chamber (1–2% O_2_). Splenic CD4^+^ and CD8^+^ T cells (> 95% purified) were co-cultured with bone marrow-derived melanoma antigen-pulsed DCs, in the presence or absence of MSCs (naïve, untreated) or TC-MSCs for 72 h with or without NLGP. Both the T cell populations were treated with NLGP only to check if NLGP has any direct role on T cell proliferation and effector function. In line with our recent report, TC-MSCs show pronounced inhibition of T cell proliferation in terms of Ki67 expression and ^3H^thymidine incorporation assay on both CD4^+^ and CD8^+^ T cells (Fig. 1). Supplementation of NLGP in this culture significantly restored the inhibition in T cell proliferation almost towards normal level in both the T cell subsets (CD4, 20 to 39%; CD8, 18 to 40%) even in the presence of TC-MSCs as measured by both Ki67 expression and thymidine incorporation assay. Given the ability of TC-MSCs to suppress the DC-induced alternate T cell functions such as IFNγ production in vitro, we next supplemented NLGP in co-culture to evaluate its corrective effect on T cell functions. NLGP supplementation significantly enhanced IFNγ secretion at late time point (72 h) from both the T cell type even in the presence of TC-MSCs. However, addition of NLGP directly on T cell population was unable to increase the basal T cell proliferation and IFNγ secretion without the presence of antigen-pulsed DCs. Therefore, our collective data suggested that NLGP is effective to reverse TC-MSC-mediated inhibitory effects on DC-induced late-phase T cell proliferation and effector IFNγ production.

### NLGP directly modulates TC-MSCs to reduce its suppressive effect on T cells

**Fig. 2:**
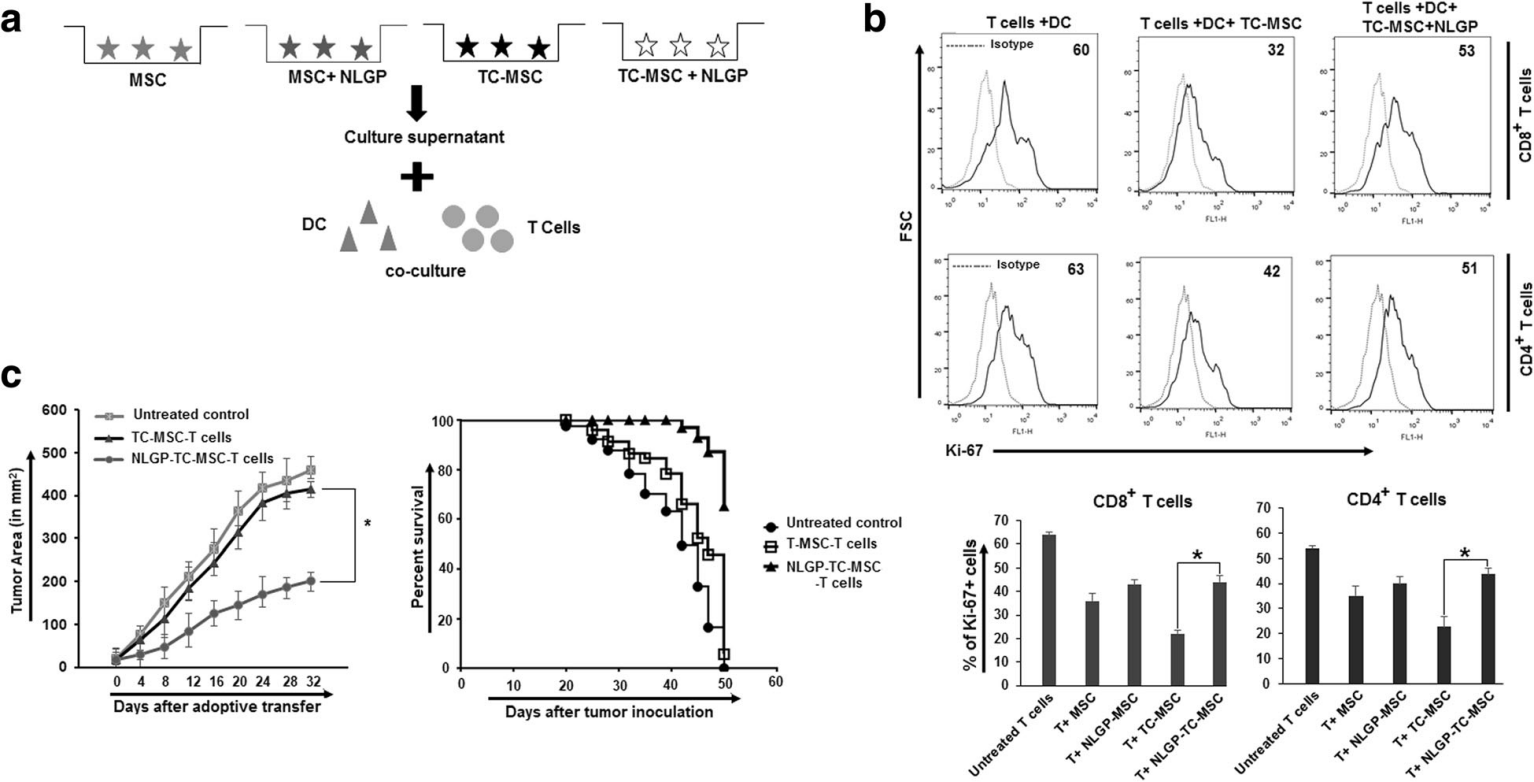
NLGP corrects TC-MSC and suppresses their inhibitory effects on T cell proliferation. **a** Diagrammatic representation of cross-talk between MSCs or TC-MSCs (NLGP treated/non-treated) and DC-T cells in co-culture. **b** Ki67 expression on CD8^+^ and CD4^+^ T cells in the presence of NLGP-treated MSC or TC-MSC culture supernatant as assessed by flow cytometry. Aggregate data obtained from six independent experiments performed. Bar diagrammatic representation of Ki67^+^CD8^+^and Ki67^+^CD4^+^T cells from MSC or TC-MSC co-culture with T cells in the presence and absence of NLGP (*n* = 6); *p < 0.05. **c** CD8^+^ T cells were cultured in the presence of TC-MSC supernatant and NLGP-treated TC-MSC supernatant for 48 h and were adoptively transferred (1 × 10^6^) through tail vein into two groups of B16 melanoma tumor bearing mice (*n* = 9, in each group) and another group of tumor-bearing mice was kept untreated as control. Mean tumor area ± SD and survivability are presented, *p < 0.05

As we found that MSC-mediated inhibition of T cell proliferation was corrected significantly by NLGP in vitro, it may be considered that NLGP could work on one or more components of the co-culture, i.e., it might modulate DCs or T cells or MSCs individually or in combination. Notably, it was already reported from our laboratory that NLGP activates T cells [13, 14] and matures immature DCs [11, 20]; thus, both of these properties can contribute to better T cell proliferation under immunosuppressive environment. Therefore, in this present work, we evaluated the modulatory effects of NLGP on TC-MSCs, and accordingly, TC-MSCs were exposed to NLGP for 48 h before initiation of DC-T cell co-culture experiments (Fig. 2a). As we already reported that TC-MSC-mediated inhibition of T cell proliferation is contact independent and secretary factor(s) dependent [9], therefore, we used cell-free culture supernatant from NLGP-treated MSCs or TC-MSCs in DC-T cell co-culture. Following 72 h, T cells were collected and their proliferation was measured by means of Ki67 assay. Addition of NLGP-treated TC-MSC supernatant shows (Fig. 2b) less suppression towards T cell proliferation than TC-MSC supernatant. So, it was clearly evident that NLGP can directly modulates TC-MSCs to reduce its inhibitory effects on T cell proliferation.

In an objective to validate the in vitro results, CD8^+^ T cells were either exposed to TC-MSC (Gr.2) or NLGP-treated TC-MSC supernatant (Gr.3) for 48 h. These cells are then injected intravenously into mice with established melanoma (average tumor volume 18 mm^2^). Obtained results clearly showed (Fig. 2c) that mice of Gr. 2 have progressive tumor (mean tumor area 416 mm^2^ on day 32; mean survival 33 days) almost similar to the group that kept untreated as control (Gr. 1, mean tumor area 445 mm^2^ on day 28). On the other hand, all the mice from Gr. 3 survived till day 50, with significantly lesser tumor load (mean tumor area 202 mm^2^). This observation further confirmed that NLGP treatment of TC-MSCs makes them unable to show their inhibitory effects on T cell functionality.

### NLGP regulates the cytokine release from TC-MSCs to restore T cell functions

**Fig. 3:**
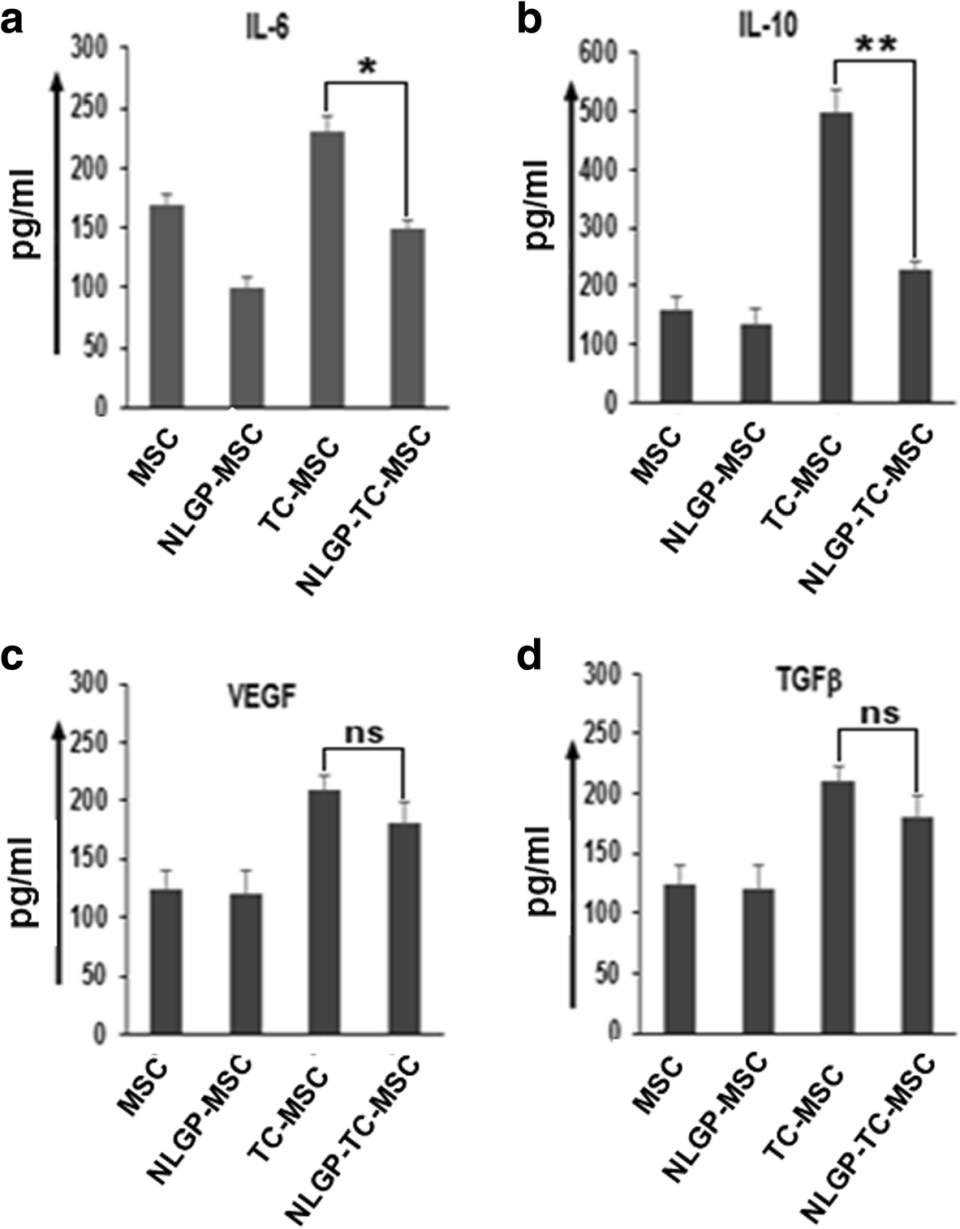
NLGP downregulates the secretion of inflammatory cytokines from TC-MSCs. MSCs and TC-MSCs were cultured in vitro in the presence or absence of NLGP for 48 h. Then, the cell-free supernatants, harvested from naive MSCs and TC-MSCs with or without NLGP treatment, were assessed for their content of **a** IL-6, **b** IL-10, **c** VEGF, and **d** TGFβ by ELISA (presented as mean ± SD values in pg/ml) using bar diagrams. Four independent experiments were performed. *p < 0.05, **p < 0.001 versus TC-MSC supernatant. ns = nonsignificant

Since the TC-MSC-mediated suppression of DC-induced T cell proliferation was found to be contact independent and secretory factor dependent [9], next, we sought to find out whether NLGP can alter the secretary profile of TC-MSCs. Cell-free supernatants were collected after 48 h from MSC and TC-MSC culture treated with or without NLGP and was quantitatively evaluated for the presence of cytokines and growth factors. The results obtained from ELISA documented that IL-10 and IL-6 were predominant in TC-MSC-derived supernatant followed by TGFβ and VEGF (Fig. 3). On the other hand, NLGP-treated MSCs showed significantly reduced secretion of IL-10 as well as IL-6, while decrease in VEGF and TGFβ is statistically insignificant. Therefore, NLGP may alter the secretory profile of TC-MSCs, which in turn resulted in restoration of T cell proliferation even in the presence of TC-MSCs.

### NLGP indirectly helps in maintaining cysteine supply to T cells from DCs

**Fig. 4:**
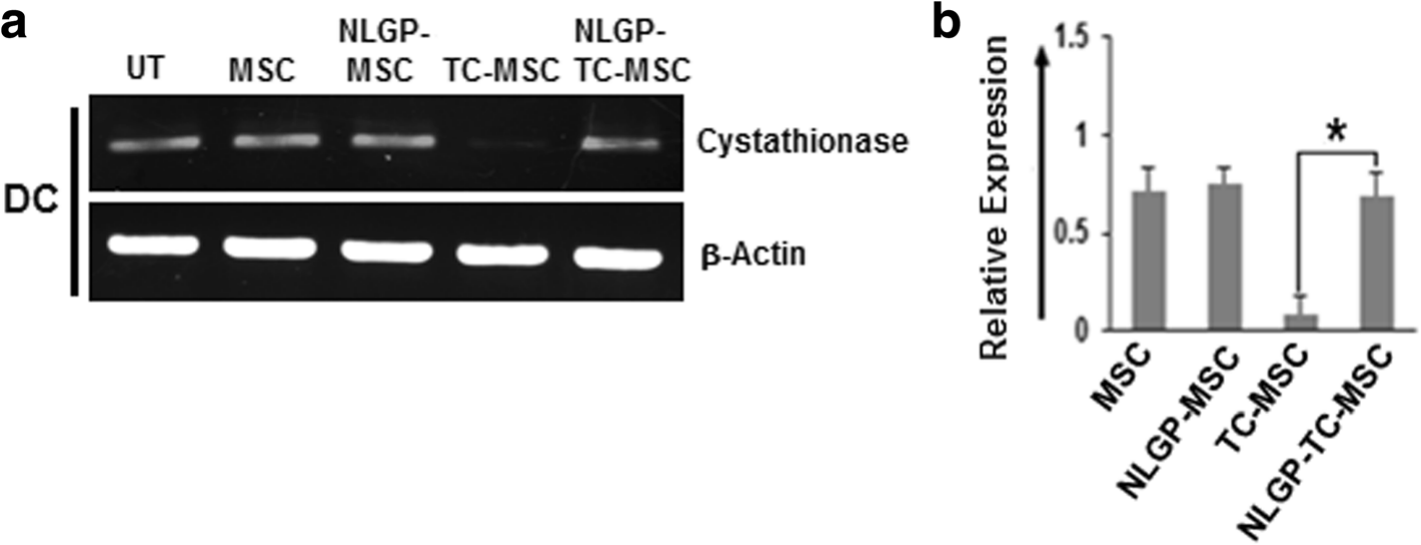
NLGP treatment of TC-MSCs restores cystathionase expression in DCs. DCs were treated in vitro with MSC, TC-MSC supernatant with or without NLGP treatment for 24 h. **a** Expression of cystathionase and β-actin mRNAs were analyzed by RT-PCR. Representative figure is presented from six independent experiments performed and **b** mean ± SD of relative expression of individual expression of target gene transcripts normalized against β-actin as presented in bar diagrams. *p < 0.05 versus DCs cultured in the presence of TC-MSC supernatant

Recently, we have also reported that TC-MSCs inhibit cystathionase expression in DCs using IL-10-STAT3 signaling pathway, thus producing cysteine scarcity to cognate T cells which eventually impedes T cell proliferation and its effector functions. As we found that NLGP was able to restore the TC-MSC-mediated inhibition of T cell proliferation remarkably, next we proceeded to check the expression of cystathionase in DCs when cultured in the presence of NLGP-treated TC-MSC supernatant in comparison to TC-MSC and MSC supernatants. The RT-PCR data showed that cystathionase expression was significantly higher in DCs, when DCs were co-cultured in the presence of NLGP-treated TC-MSC supernatant than the TC-MSC supernatant only (Fig. 4). Thus, collective data is suggestive that NLGP may modulate TC-MSC supernatant in such a manner that it became unable to show its inhibitory effect on cystathionase gene expression in DCs. Therefore, in the presence of NLGP-treated TC-MSC supernatant, DCs can export out sufficient amount of cysteine in extracellular environment for the cognate T cells to proliferate optimally.

### NLGP regulates IL-10-STAT3 signaling to withdraw MSCs inhibition

**Fig. 5:**
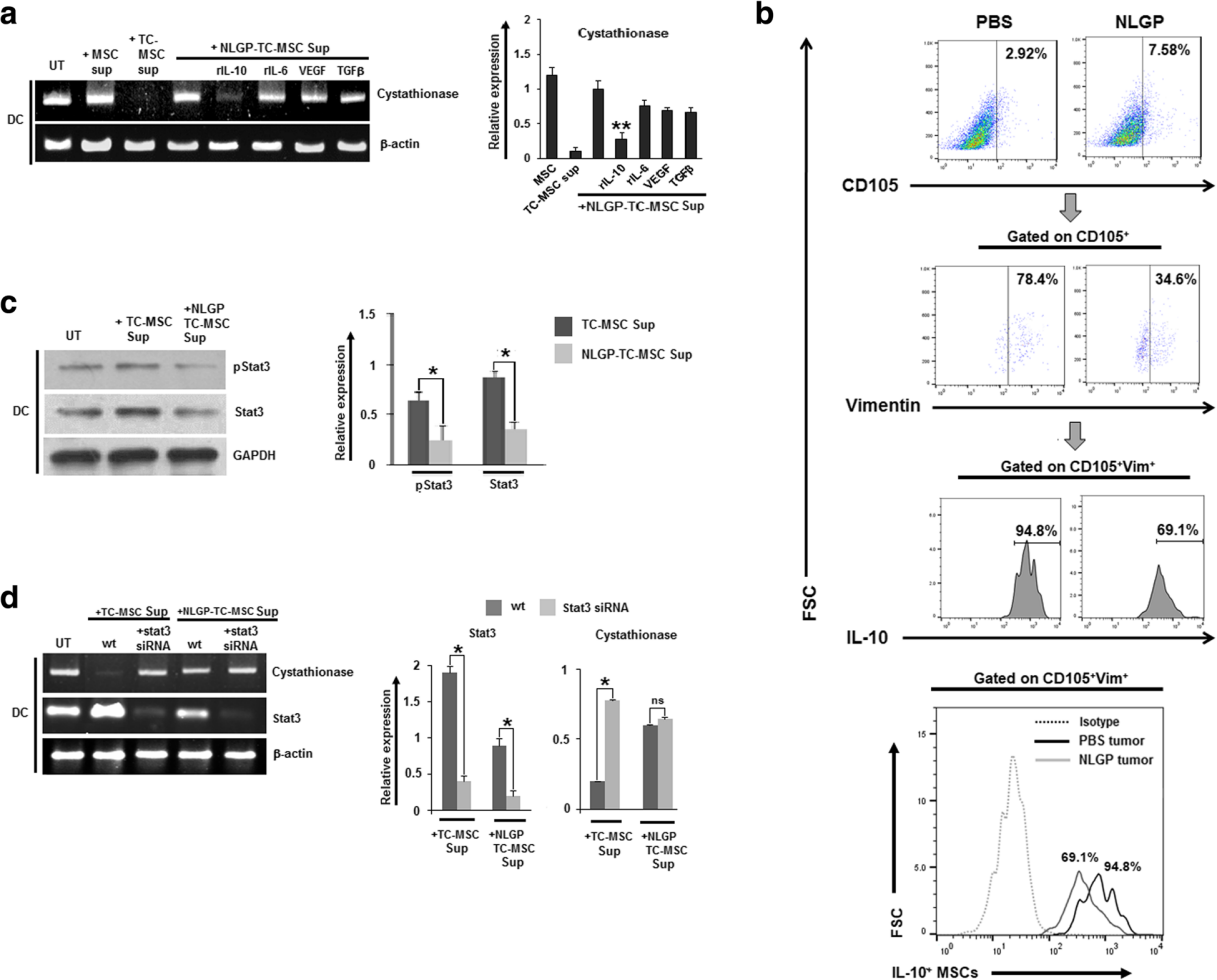
NLGP withdraws TC-MSC-mediated inhibition of cystathionase expression in DCs by regulating IL-10/STAT3 signaling pathway. **a** DCs were treated in vitro with normal MSC, TC-MSC, and NLGP-treated TC-MSC supernatant along with equal concentration of rIL-10, rIL-6, rVEGF, and rTGFβ respectively. Expression of cystathionase and β-actin mRNAs was analyzed by RT-PCR. Representative figures are presented from three independent experiments performed with the relative expression of target gene transcripts normalized against β-actin transcript levels as presented in bar diagrams. **p < 0.001 versus DCs cultured in the presence of NLGP-treated TC-MSC supernatant. **b** The expression of CD105^+^Vimentin^+^IL-10^+^ MSCs in tumors isolated from PBS and NLGP-treated tumor-bearing mice respectively is depicted in representative histogram. Gating strategy with FMO are shown in Additional file 1: Figure S1. **c** DCs were exposed to TC-MSC versus NLGP-treated TC-MSC culture supernatants and their expression levels of pSTAT3 and STAT3 were evaluated by western blot. Representative figures are presented from three different experiments. Mean relative expression of pSTAT3 and STAT3 are also presented in each group of DCs normalized against GAPDH. *p < 0.05; **d** DCs were pre-treated in vitro with stat3 siRNA before being exposed to TC-MSC as well as NLGP-treated TC-MSC supernatant and the expression levels of cystathionase and β-actin mRNA determined by RT-PCR. Representative figures are presented from three independent experiments performed with the relative expression of target gene transcripts normalized against β-actin mRNA as presented in bar diagram. *p < 0.05. ns = nonsignificant

Given the facts that the inhibition of T cell proliferation by TC-MSC was secretory factor dependent and all the prevalent factors present in TC-MSC supernatant, i.e., IL-10 along with IL-6, VEGF, and TGFβ were downregulated by NLGP, next, we investigated to find out the key cytokine, which was being downregulated by NLGP in TC-MSC supernatant to restore the cystathionase expression in DCs. Accordingly, DCs were exposed to TC-MSC supernatant with or without NLGP treatment along with corresponding recombinant proteins of each cytokine that was followed by the assessment of the expression of cystathionase. Notably, addition of recombinant IL-10 in NLGP-treated TC-MSC supernatant shows complete abrogation of cystathionase expression in DCs (Fig. 5a). However, cystathionase expression was almost normal in DCs in NLGP-treated TC-MSC supernatant when compared to the cystathionase expression in the presence of TC-MSC supernatant. So, it was evident that NLGP inhibits the IL-10 secretion from TC-MSCs which resulted in almost normal expression of cystathionase enzyme in DCs making them competent to export sufficient amount of cysteine to T cells for their proliferation.

To check the efficacy of NLGP in regulating the number of IL-10-secreting MSCs in vivo, two groups of mice with established B16 melanoma tumor on around the 7th day of inoculation were either administered with NLGP (25 μg/mice) or PBS weekly for 4 weeks in total. Then, the tumors were harvested from each group, and the status of CD105^+^Vimentin^+^IL-10^+^ MSCs was checked by flow cytometry (Fig. 5b). It was observed that the number of IL-10-secreting MSCs is significantly lower in tumor from NLGP-treated mice than tumor from mice with PBS treatment.

It has been well established that STAT3 is an obligate factor required to mediate the effect of IL-10, and our previous study revealed that TC-MSC supernatant induce STAT3 phosphorylation, followed by its nuclear localization to bind with GAS motif present within cystathionase promoter [9]. Therefore, to check the effect of NLGP on STAT3, DCs were cultured in the presence of either NLGP-treated or untreated TC-MSC supernatant and the expression of STAT3 and pSTAT3 was studied. Interestingly, pSTAT3 expression was downregulated in DCs in the presence of NLGP-treated TC-MSC supernatant in comparison to the DCs exposed to TC-MSC supernatant (Fig. 5c). Therefore, TC-MSC-secreted factor(s) activate/phosphorylate STAT3. However, supplementation of NLGP-treated TC-MSC supernatant in DC culture caused prominent downregulation in Stat3 phosphorylation suggesting alteration of TC-MSC-secreted factor(s) by NLGP. To further validate the effect of NLGP on STAT3, DCs were subjected to knockdown for STAT3 using respective SiRNA and cultured with supernatant isolated from NLGP-treated TC-MSCs and TC-MSCs only. Cystathionase expression was almost normal in Stat3-silenced DCs; those were expanded in either TC-MSC or NLGP-treated TC-MSC supernatant establishing the fact that NLGP modulates IL-10/Stat3 signaling pathway to diminish the inhibitory effect of TC-MSCs towards cystathionase expression (Fig. 5d).

## Discussion

As tumors employ many untransformed cells during cancer progression, many researchers naturally explored the contribution of endogenous stem cells to cancers. A growing body of evidence suggests that MSCs derived from both local (e.g., adipose tissue) and distant (e.g., bone marrow) sources contribute to the tumor stroma. MSCs home to progressively growing tumors in large numbers, where they aggravate cancer cell proliferation, motility, invasion, and metastasis, nurture angiogenesis and suppress antitumor immune responses. We have discovered recently a novel mechanism for suppressed activity of CD8^+^ T cells by tumor-associated MSCs (TC-MSCs). Precisely, we revealed that TC-MSC-secreted IL-10 represses cystathionase gene expression in DCs and thereby inhibits cysteine production to export it to neighboring T cells for their optimum proliferation [9]. On the other hand, we have reported earlier that NLGP restricts the growth of murine sarcoma and melanoma significantly in CD8^+^ T cell-dependent manner [13–15]. In that case, NLGP fosters antigen presentation (SarAg or MelAg) to CD8^+^T cells by influencing APCs [11, 21]. We have also reported that NLGP-mediated normalization of tumor microenvironment (TME) allows T cells to function optimally that help to inhibit the tumor growth. This observation encouraged us to find out the direct influence of tumor “educated” MSCs on such T cells in vitro and to explore the effect of NLGP on MSC-T cell crosstalk within TME. Here, in this study, we elucidated that (i) NLGP significantly inhibits the immunosuppression of tumor MSCs and restores T cell effector functions like normal, (ii) NLGP reduces the IL-10 secretion by tumor MSCs, and finally, (iii) the decreased IL-10 secretion results in reduced phosphorylation of STAT3 leading to transcription of cystathionase gene in DCs. Consequently, DCs are able to export out sufficient amount of cysteine to the cognate T cells required for their optimum proliferation and late-phase effector function.

To mimic the functionality of tumor-associated MSCs as closely as possible in vitro, we conditioned normal MSCs with B16 melanoma tumor supernatant and cultured them in hypoxic chamber (1–2% O_2_) which are referred here as TC-MSCs. We co-cultured these TC-MSCs with purified CD8^+^ and CD4^+^ T cells in the presence or absence of NLGP keeping naïve T cells and normal MSCs as control. In agreement with our previous report, we found TC-MSCs severely suppress DC-induced T cell proliferation and late-phase effector functions. As a unique modulator of immune functionality, NLGP was also found to be effective to reduce MSCs’ suppression on T cell proliferation (detected by ^3^H incorporation, Ki67 assay) and restore IFNγ secretion significantly. Our results also clearly demonstrated that NLGP modifies TC-MSCs to inhibit their immunosuppressive effect on DC-induced T lymphocyte proliferation. Notably, it was already reported from our laboratory that NLGP activates T cells [13] and matures DCs [11] in tumor condition in vivo.

We already reported that TC-MSCs exhibit their inhibitory effects on T cell functionality in contact independent and IL-10-dependent manner [9], and here, in this study, we proved that NLGP decreased the IL-10 expression in TC-MSCs to a substantial extent. We also reported that TC-MSC-secreted IL-10 represses cystathionase gene expression in DCs and thereby inhibits cysteine production to export it to neighboring T cells for their optimum proliferation. It is worth mentioning in this context that cysteine is an essential amino acid for T cells required for their proliferation and cytotoxicity [22] as they are unable to synthesize it by their own [23]. Hence, T cells are completely dependent on extracellular cysteine provided by APCs in the surrounding environment [24–26]. This study revealed that NLGP indirectly helps to maintain almost normal level of cystathionase gene expression in DCs under the influence of TC-MSCs by diminishing their IL-10 production. Our previous publication also ascertained that binding of IL-10 with IL-10R present on DC surface leads to Stat3 phosphorylation and this phosphorylated Stat3 binds with the cystathionase promoter and represses its transcription. Here, we also observed that the level of Stat3 phosphorylation was declined significantly in DCs, when exposed with NLGP-treated TC-MSC supernatant with respect to the DCs treated with TC-MSC supernatant. This observation corroborates the fact that NLGP inhibited the IL-10 secretion from TC-MSCs which in turn resulted in lesser Stat3 phosphorylation that ultimately helps in almost normal transcription of cystathionase gene in DCs. Therefore, DCs become compatible to supply adequate cysteine to the cognate T cells required for their optimal effector functions.

In connection to the in vivo anti-tumor activity of CD8^+^ T cells, such cells were expanded to TC-MSC supernatant and injected intravenously to solid tumor-bearing mice. This treatment causes non-functionality of the CD8^+^ T cells. Interestingly, such non-functionality was rescued due to expansion of same cells to NLGP-treated TC-MSCs. Observed NLGP activity might be due to the downregulation of IL-10/STAT3 hyperactivity causing suppression of cystathionase gene expression in DCs. Our data documented that NLGP may act as a potential immunotherapeutic drug that modify highly immunosuppressive TC-MSCs by downregulating their IL-10-secreting properties in tumor microenvironment. IL-10 as an immunoregulatory cytokine inhibits antigen-specific T cell responses by impeding antigen presentation and co-stimulation [27]. In this context, it is worthy to mention here that in TME, there are huge number of IL-10-secreting cell populations (MDSCs, TAMs) though IL-10^+^ MSCs are predominating among them [9]. Therefore, NLGP could be a potential immunotherapeutic drug to inhibit the dialog between tumor stroma and immune cells besides reinforcing its powerful efficacy against IL-10-mediated immunosuppression that ultimately reestablishes the optimum T cell functions of anti-tumor nature.

## Conclusions

We demonstrated NLGP, a natural nontoxic immunomodulator, modifies TC-MSCs by reducing their IL-10 secretion that ultimately results in reduced phosphorylation of STAT3 leading to transcription of cystathionase gene in DCs. Consequently, DCs are able to export out sufficient amount of cysteine to the cognate T cells required for their optimum proliferation and late-phase effector functions. Our findings also established NLGP as a prospective immunotherapeutic agent to control the functions and behavior of TC-MSCs in TME providing an important new approach that might be effective in cancer treatment.

## Additional files

Additional file 1: **Figure S1.** Gating strategy of CD105^+^Vimentin^+^IL-10^+^ MSCs, as prescribed in Fig. 5b, are shown along with their respective FMO for both PBS and NLGP groups. (PNG 314 kb)

Additional file 2: **Table S1.** List of antibodies used with their respective manufacturers, catalog numbers, clones, hosts and isotypes. (DOCX 15 kb)

## Data Availability

Data sharing is not applicable to this article as no datasets were generated or analyzed during the current study.

## Abbreviations

APCs: Antigen-presenting cells
BM: Bone marrow
BMDC: Bone marrow-derived dendritic cell
ConA: Concanavalin A
FBS: Fetal bovine serum
IFNγ: Interferon-γ
IL-10: Interleukin-10
IL-2: Interleukin-2
IL-4: Interleukin-4
IL-6: Interleukin-6
LDH: Lactate dehydrogenase
LPS: Lipopolysaccharide
MACS: Magnetic activated cell sorting
MelAg: Melanoma antigen
MHC-II: Major histocompatibility complex-II
MSCs: Mesenchymal stem cells
NK: Natural killer cell
NLGP: Neem leaf glycoprotein
rm-GMCSF: Recombinant mouse-granulocyte macrophage colony stimulating factor
SarAg: Sarcoma antigen
STAT3: Signal transducer and activator of transcription 3
TGF-β: Transforming growth factor beta
TME: Tumor microenvironment
VEGF: Vascular endothelial growth factor

## References

[1] Egeblad M, Nakasone ES, Werb Z (2010). Tumors as organs: complex tissues that interface with the entire organism. Dev Cell.

[2] Bussard KM, Mutkus L, Stumpf K, Gomez-Manzano C, Marini FC (2016). Tumor-associated stromal cells as key contributors to the tumor microenvironment. Breast Cancer Res.

[3] Djouad F, Plence P, Bony C, Tropel P, Apparailly F, Sany J (2003). Immunosuppressive effect of mesenchymal stem cells favors tumor growth in allogeneic animals. Blood..

[4] Rasmusson I, Ringden O, Sundberg B, Le Blanc K (2005). Mesenchymal stem cells inhibit lymphocyte proliferation by mitogens and alloantigens by different mechanisms. Exp Cell Res.

[5] Beyth S, Borovsky Z, Mevorach D, Liebargall M, Gazit Z, Aslan H (2005). Human mesenchymal stem cells alter antigen-presenting cell maturation and induce T-cell unresponsiveness. Blood..

[6] Sotiropoulou PA, Perez SA, Gritzapis AD, Baxivanis CM, Papamichail M (2006). Interactions between human mesenchymal stem cells and natural killer cells. Stem Cells.

[7] Corcione A, Benvenuto F, Ferretti E, Giunti D, Cappiello V (2006). Cazzantiet al. Human mesenchymal stem cells modulate B-cell functions. Blood..

[8] Luz-Crawford P, Kurte M, Bravo-Alegría J, Contreras R, Nova-Lamperti E, Tejedor G (2013). Mesenchymal stem cells generate a CD4+CD25+Foxp3+ regulatory T cell population during the differentiation process of Th1 and Th17 cells. Stem Cell Res Ther.

[9] Ghosh T, Barik S, Bhuniya A, Dhar J, Dasgupta S, Ghosh S (2016). Tumor-associated mesenchymal stem cells inhibit naïve T cell expansion by blocking cysteine export from dendritic cells. Int J Cancer.

[10] Chakraborty Krishnendu, Bose Anamika, Pal Smarajit, Sarkar Koustav, Goswami Shyamal, Ghosh Diptendu, Laskar Subrata, Chattopadhyay Utpala, Baral Rathindranath (2008). Neem leaf glycoprotein restores the impaired chemotactic activity of peripheral blood mononuclear cells from head and neck squamous cell carcinoma patients by maintaining CXCR3/CXCL10 balance. International Immunopharmacology.

[11] Goswami S, Bose A, Sarkar K, Roy S, Chakrabarty T, Sanyal U (2010). Neem leaf glycoprotein matures myeloid derived dendritic cells and optimizes anti-tumor T cell functions. Vaccine..

[12] Sarkar Koustav, Goswami Shyamal, Roy Soumyabrata, Mallick Atanu, Chakraborty Krishnendu, Bose Anamika, Baral Rathindranath (2010). Neem leaf glycoprotein enhances carcinoembryonic antigen presentation of dendritic cells to T and B cells for induction of anti-tumor immunity by allowing generation of immune effector/memory response. International Immunopharmacology.

[13] Mallick A, Barik S, Goswami KK, Banerjee S, Ghosh S, Sarkar K (2013). Neemleaf glycoprotein activates CD8+ T cells to promote therapeutic anti-tumor immunity inhibiting the growth of mouse sarcoma. PLoS One.

[14] Barik S, Banerjee S, Mallick A, Goswami KK, Roy S, Bose A (2013). Normalization of tumor microenvironment by neem leaf glycoprotein potentiates effector T cell functions and therapeutically intervenes in the growth of mouse sarcoma. PLoS ONE.

[15] Barik S, Banerjee S, Sarkar M, Bhuniya A, Roy S, Bose A (2015). Neem leaf glycoprotein optimizes effector and regulatory functions within tumor microenvironment to intervene therapeutically the growth of B16 melanoma in C57BL/6 mice. Trials Vaccinol.

[16] Chakraborty Krishnendu, Bose Anamika, Chakraborty Tathagata, Sarkar Koustav, Goswami Shyamal, Pal Smarajit, Baral Rathindranath (2010). Restoration of dysregulated CC chemokine signaling for monocyte/macrophage chemotaxis in head and neck squamous cell carcinoma patients by neem leaf glycoprotein maximizes tumor cell cytotoxicity. Cellular & Molecular Immunology.

[17] Lowry OH, Rosenbrough NJ, Farr AL, Randall RJ (1951). Protein measurement with the Folin phenol reagent. J BiolChem.

[18] Bose A, Taylor JL, Alber S, Watkins SC, Garcia JA, Rini BI (2011). Sunitinib facilitates the activation and recruitment of therapeutic anti-tumor immunity in concert with specific vaccination. Int J Cancer.

[19] Goswami Kuntal Kanti, Sarkar Madhurima, Ghosh Sarbari, Saha Akata, Ghosh Tithi, Guha Ipsita, Barik Subhasis, Banerjee Saptak, Roy Soumyabrata, Bose Anamika, Dasgupta Parthasarathi, Baral Rathindranath (2016). Neem leaf glycoprotein regulates function of tumor associated M2 macrophages in hypoxic tumor core: Critical role of IL-10/STAT3 signaling. Molecular Immunology.

[20] Sarkar K, Bose A, Chakraborty K, Haque E, Ghosh D, Goswami S (2008). Neem leaf glycoprotein helps to generate carcinoembryonic antigen specific anti-tumor immune responses utilizing macrophage-mediated antigen presentation. Vaccine..

[21] Levring TB, Hansen AK, Nielsen BL, Kongsbak M, von Essen MR, Woetmann A (2012). Activated human CD4+ T cells express transporters for both cysteine and cysteine. Sci Rep.

[22] Bannai S (1984). Transport of cystine and cysteine in mammalian cells. Biochim Biophys Acta.

[23] Sato H, Watanabe H, Ishii T, Bannai S (1987). Neutral amino acid transport in mouse peritoneal macrophages. J Biol Chem.

[24] Angelini G, Gardella S, Ardy M, Ciriolo MR, Filomeni G, Di Trapani G (2002). Antigen-presenting dendritic cells provide the reducing extracellular microenvironment required for T lymphocyte activation. Proc Natl Acad Sci USA.

[25] Castellani P, Angelini G, Delfino L, Matucci A, Rubartelli A (2008). The thiol redox state of lymphoid organs is modified by immunization: role of different immune cell populations. Eur J Immunol.

[26] Demangel C, Bertolino P, Britton WJ (2002). Autocrine IL-10 impairs dendritic cell (DC)-derived immune responses to mycobacterial infection by suppressing DCtrafficking to draining lymph nodes and local IL-12 production. Eur J Immunol.

[27] Knolle, Uhrig, Hegenbarth, LOser, Schmitt, Gerken, Lohse (1998). IL-10 down-regulates T cell activation by antigen-presenting liver sinusoidal endothelial cells through decreased antigen uptake via the mannose receptor and lowered surface expression of accessory molecules. Clinical and Experimental Immunology.

