# Supplementary figures and images for: NLGP counterbalances the immunosuppressive effect of tumor-associated mesenchymal stem cells to restore effector T cell functions
Source: Stem Cell Res Ther. 2019 Sep 23;10:296. doi: 10.1186/s13287-019-1349-z (PMC6757425; doi:10.1186/s13287-019-1349-z)

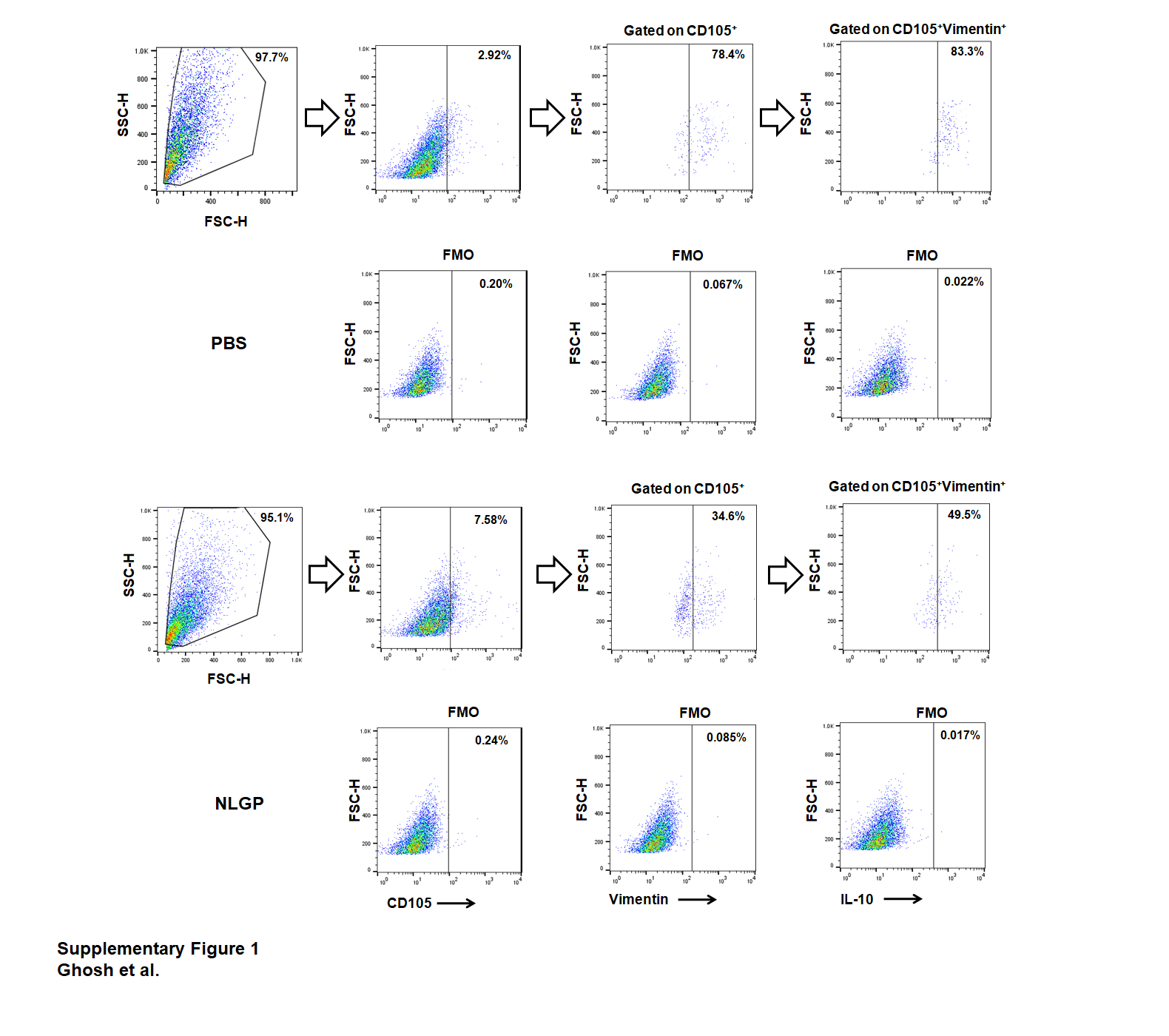

Supplement: Supplementary file 1 — Figure S1. Gating strategy of CD105+Vimentin+IL-10+ MSCs, as prescribed in Fig. 5b, are shown along with their respective FMO for both PBS and NLGP groups. (PNG 314 kb) [file 13287_2019_1349_MOESM1_ESM.png]
