# Supplementary material for: NLGP counterbalances the immunosuppressive effect of tumor-associated mesenchymal stem cells to restore effector T cell functions
Source: Stem Cell Res Ther. 2019 Sep 23;10:296. doi: 10.1186/s13287-019-1349-z (PMC6757425; doi:10.1186/s13287-019-1349-z)
Supplement: Supplementary file 2 — Table S1. List of antibodies used with their respective manufacturers, catalog numbers, clones, hosts and isotypes. (DOCX 15 kb) [file 13287_2019_1349_MOESM2_ESM.docx]

**Table S1. List of Antibodies used**

| **Names** | **Manufacturer** | **Catalog No.** | **Antibody clones** | **Host/Isotype** | **Fluorochrome** |
| --- | --- | --- | --- | --- | --- |
| Anti m-CD105 | eBiosciences | 12-1051-81 | MJ7/18 | Rat / IgG2a, kappa | PE |
| Anti m-IFNγ | eBiosciences | 14-7312-85 | R4-6A2 | Rat / IgG1, kappa | Purified |
| Anti m-IL-10 | Biolegend | 505001 | JES5-16E3 | Rat / IgG2b, kappa | Purified |
| Anti m-IL-6 | Biolegend | 504501 | MP5-20F3 | Rat / IgG1, kappa | Purified |
| Anti m-Ki-67 | BD Biosciences | 652401 | 16A8 | Rat / IgG2a, kappa | Purified |
| Anti m-STAT3  (STAT3 Sampler Kit) | BD Transduction laboratories | 519002095 | - | Goat/ IgG | Purified |
| Anti m-pSTAT3 | BD Biosciences | 612357 | 4/P-STAT3 | Mouse / IgG2a, kappa | Purified |
| Anti m-TGFβ | BD Biosciences | 555052 | A75-2 | Rat / IgG2a, kappa | Purified |
| Anti m-VEGF | Santa Cruz Biotechnology | sc-507 | 147 | Rabbit / IgG | Purified |
| Anti m-vimentin | BD Biosciences | 550513 | RV202 | Mouse / IgG1 | Purified |
| BD iMAG Anti-Mouse CD4 Magnetic Particles- DM | BD Biosciences | 551539 | GK1.5 | Rat/ IgG1 | NA |
| BD iMAG Anti-Mouse CD8a Particles- DM | BD Biosciences | 551516 | 53-6.7 | Rat/ IgG1 | NA |
| Secondary anti mouse FITC | Sigma | F0257-5ml | Polyclonal | Mouse/ IgG | Peroxidase cojugated |
| Anti rat HRP | Gene i | 62114148001A | Polyclonal | Rat/IgG | Peroxidase cojugated |
| Anti mouse HRP | Sigma | A5278- 1ml | Polyclonal | Mouse/ IgG | Peroxidase cojugated |
| Anti rabbit HRP | Sigma | A0545-1Ml | Polyclonal | Rabbit/ IgG | Peroxidase cojugated |
| Anti rat FITC | Sigma | F1763 -5ml | Polyclonal | Rat/ IgG1 | FITC conjugated |
| Anti rat PE Cy5.5 | Abcam | Ab130803- 1ml | Polyclonal | Rat/ IgG | PE cy5.5 |
